# Circulating microRNAs as Potential Biomarkers in Non-Alcoholic Fatty Liver Disease and Hepatocellular Carcinoma

**DOI:** 10.3390/jcm5030030

**Published:** 2016-03-03

**Authors:** Marta B. Afonso, Pedro M. Rodrigues, André L. Simão, Rui E. Castro

**Affiliations:** 1 Research Institute for Medicines (iMed.ULisboa), Faculty of Pharmacy, Universidade de Lisboa, 1649-003 Lisbon, Portugal; mbafonso@ff.ulisboa.pt (M.B.A.); pmvrsrodrigues@ff.ulisboa.pt (P.M.R.); adlsimao@ff.ulisboa.pt (A.L.S.); 2 Department of Biochemistry and Human Biology, Faculty of Pharmacy, Universidade de Lisboa, 1649-003 Lisbon, Portugal

**Keywords:** microRNA, nonalcoholic fatty liver disease, hepatocellular carcinoma, biomarker, serum, plasma, liver

## Abstract

Obesity and metabolic syndrome are growing epidemics worldwide and greatly responsible for many liver diseases, including nonalcoholic fatty liver disease (NAFLD). NAFLD often progresses to cirrhosis, end-stage liver failure and hepatocellular carcinoma (HCC), the most common primary liver cancer and one of the leading causes for cancer-related deaths globally. Currently available tools for the diagnosis of NAFLD staging and progression towards HCC are largely invasive and of limited accuracy. In light of the need for more specific and sensitive noninvasive molecular markers, several studies have assessed the potential of circulating microRNAs (miRNAs) as biomarkers of liver injury and hepatocarcinogenesis. Indeed, extracellular miRNAs are very stable in the blood, can be easily quantitated and are differentially expressed in response to different pathophysiological conditions. Although standardization procedures and larger, independent studies are still necessary, miRNAs constitute promising, clinically-useful biomarkers for the NAFLD-HCC spectrum.

## 1. Introduction

The burden of liver diseases is rapidly growing and already represents a major health concern worldwide. In particular, nonalcoholic fatty liver disease (NAFLD) already represents the most common chronic liver disease [1]. There is no current pharmacological treatment for NAFLD; in obese patients, bariatric surgery and lifestyle interventions for weight loss remain the cornerstone of NAFLD treatment. If untreated, patients with simple steatosis often progress to more severe stages of NAFLD, namely nonalcoholic steatohepatitis (NASH). In turn, NASH constitutes a predisposing factor for the development of liver cirrhosis and hepatocellular carcinoma (HCC), with liver cancer representing the sixth most common cancer and the third leading cause of cancer-related death [2]. In fact, and unlike with other cancers, the mortality rate of patients with HCC is very close to the incidence rate due to the chemo-resistant nature of the tumor and because its management is complicated by the high prevalence of liver cirrhosis and late-stage diagnosis. Further, the ability to predict HCC development in chronic patients is very difficult and represents one of the major challenges at the present time [3].

Liver biopsy remains one of the few reliable methods to distinguish steatosis from NASH and to identify the extent of liver injury and fibrosis [4]. However, it represents an invasive method and often is associated with severe complications. In an attempt to use non-invasive methods of diagnosis, a mild to moderate increase in serum aminotransferases, with a (aspartate aminotransferase) AST/(alanine aminotransferase) ALT ratio <1 has been suggested as a good predictor of NAFLD, in the absence of advanced fibrosis [5]. Still, normal ALT levels can be found in NAFLD patients representing the entire spectrum of disease severity, from simple steatosis to advanced NASH [6]. Similarly, measurement of serum alpha-fetoprotein (AFP) has long been proposed as a diagnostic marker of HCC, but it fails to diagnose approximately one-third of patients with early-stage HCC and is also found elevated in benign liver diseases, such as hepatitis and cirrhosis [7]. As such, there is an urgent need for the identification of novel and more specific noninvasive biomarkers that can facilitate the early diagnosis of NAFLD and HCC, monitoring disease progression, prognosis and treatment response.

miRNAs play an important role in regulating gene expression and, importantly, are released into the extracellular space and body fluids, where they remain remarkably stable. As such, circulating miRNAs have been investigated in a wide variety of NAFLD and HCC animal models, as well as in a large cohort of patients, holding great potential as robust biomarkers; identifying disease progression-driven alterations in circulating miRNAs could be a novel minimally-invasive means for the diagnosis and prognosis of the NAFLD to HCC transition.

## 2. Non-Coding RNAs as Biomarkers

Non-coding RNAs have been implicated in the regulation of almost all cell processes, through diverse biological functions. Conversely, their deregulation is implicated in the pathogenesis of several diseases, including liver diseases, highlighting the relevance of its therapeutic targeting. According to their size, non-coding RNAs can be grouped into two subfamilies: small non-coding RNAs, up to 200 bases, and long non-coding RNAs (lncRNAs), over 200 bases [8]. Notably, both small and long non-coding RNAs play a role in both transcriptional and translational control of liver gene expression. In the last decade, nucleic acids have been extensively studied regarding their potential role as biomarkers. Their advantages rely on a technically simple detection and amplification, as well as on the fact that these molecules are much less affected by degradation and/or modification when compared to other markers, such as proteins or carbohydrates [8].

### 2.1. Long Non-Coding RNAs

lncRNAs exert their functional effect by binding to a complementary sequence of their target genes, resulting in reduced gene expression [9], or by sequestering histone modification proteins or transcription promoters, thus influencing the expression of the respective target genes. Of note, lncRNAs may also interact with miRNAs [10].

Little is yet known about the role of lncRNAs in NAFLD. Still, a recent study demonstrated that amelioration of NAFLD by berberine, on an *in vivo* model of disease, involved the modulation of 538 lncRNAs [11]. In particular, *MRAK052686*, a conserved lncRNA, correlated with downregulated nuclear factor (erythroid-derived 2)-like 2 (Nrf2) levels in the steatotic liver, both of which were reversed by berberine administration. The functional role of lncRNAs in liver cancer is also being reported [12,13]; Panzitt *et al.* identified hepatocyte-specific lncRNA highly up-regulated in liver cancer (HULC) as upregulated 33-fold in human samples of HCC tissues, compared to normal samples [14], suggesting its role as an oncogene lncRNA. Indeed, lncRNAs appear to mostly act as oncogenes in the liver; lncRNA H19 expression is highly increased in patients with hepatitis B virus (HBV)-induced HCC [15], and the newly-identified lncRNA high expression in HCC(HEIH) has been suggested to play a pivotal role in liver carcinogenesis, as its expression is significantly increased in HCC patients, particularly those with liver cirrhosis [16].

Not many studies have addressed the role of serum lncRNAs as liver disease biomarkers. Still, Lu and coworkers reported that several lncRNAs are increased in the serum of HCC and HBV patients, compared to controls [17]. As such, it may be that lncRNAs may function as predictive biomarkers in serum. This exciting new filed of research will undoubtedly continue to grow in upcoming years, and lncRNAs may one day constitute an important new resource to identify new biomarkers and therapeutic molecular targets for liver diseases.

### 2.2. Small Non-Coding RNAs

sncRNAs may be classified as small nucleolar RNAs (snoRNAs), small nuclear RNAs (snRNAs), Piwi-RNAs (piRNAs) and miRNAs. snoRNAs play an active role in the formation of small nucleolar ribonucleoproteins. Specifically, snoRNAs act by guiding ribosomal and some spliceosomal RNAs for post-transcriptional modifications [18,19]. Few have yet been identified as circulating biomarkers, although Liao *et al.* recently reported that snoRNA SNORD33/66/76 might serve as a plasma biomarker to diagnose non-small-cell lung cancer [20]. snRNAs are usually found within the splicing speckles of the nucleus, and their main function is the guidance of pre-mRNAs [21]. U2 snRNA has been proposed as biomarker for melanoma, ovarian, pancreatic, lung and colorectal cancer [22,23,24,25], although more studies are needed to clarify and determine its applicability as a diagnostic molecule. piRNAs interact with Piwi proteins, a subgroup of argonaute (Ago) proteins, and maintain genomic integrity exerted in germline stem cells. Of note, piRNAs were recently detected in human saliva [26], raising the question of whether piRNAs might serve as biomarkers.

miRNAs constitute, undoubtedly, the best studied sncRNAs involved in liver disease progression or serving as biomarkers of liver disease (reviewed in [27]). miRNAs represent a group of evolutionarily-conserved sncRNAs, with 21–25 nucleotides, that post-transcriptionally regulate gene expression [28]. Because miRNAs may regulate the expression of several proteins and modulate several pathways and biological processes, such as cell death, proliferation and differentiation, their deregulation strongly correlates with different diseases [29,30,31].

The knowledge of miRNA function has been growing exponentially; in particular, a strong effort is being made to identify miRNAs that may serve as circulating biomarkers, allowing for the early diagnosis of different pathologies. Apart from blood/plasma, miRNAs have been detected in many different biofluids, such as amniotic fluid, breast milk, bronchial lavage, cerebrospinal and peritoneal fluid, tears, saliva, urine, bile and seminal fluid [32,33,34]. Given the existence of well-known and established methods to quantify their expression, as well as their tissue specificity [35,36], miRNAs are prime candidates to be established as noninvasive diagnostic and prognostic biomarkers. Of note, particularly for cancer, they may also be used to predict and monitor the efficacy of anticancer therapies, namely response to treatment and drug resistance [37].

## 3. Analysis of Circulating miRNAs

Extracellular miRNAs are found in all mammalian body fluids. In blood, miRNAs are remarkably stable and less susceptible to nuclease degradation when compared to mRNAs [36,38,39,40,41]. For instance, incubation of plasma samples at room temperature for up to 24 hours has minimal effects on the levels of circulating miRNAs. In addition, miRNAs remain relatively stable in plasma samples even after multiple freeze-thaw cycles. In contrast, spike-in miRNAs added to plasma are rapidly degraded [36,42]. This is due, at least in part, to their small size and because miRNAs circulate either encapsulated in extracellular vesicles (EVs) or in association with proteins, mainly Ago2 [38]. Other members of the Ago family, namely Ago1, Ago3 and Ago4, can also associate with extracellular miRNAs, with different tissue specificities [38,43]. Circulating miRNAs have also been found associated with RNA binding protein nucleophosmin (NPM1), which also provides protection from miRNA degradation [40]. miRNAs are further transported in plasma by high-density lipoproteins (HDL), which are capable of entering target cells for miRNA transcription, while avoiding lysosomal degradation [39]. Binding between miRNAs and lipoproteins is not yet well understood, although some studies suggest that miRNAs associate with lipoproteins when entering blood circulation, rather than being packaged in HDL and low density lipoprotein (LDL) particles within the cell [39]. The association of miRNAs with lipoproteins also shields them from degradation by RNases. In EVs, it is the existence of a lipid layer surrounding the miRNA cargo that protects it from destruction. Still, it may be that extracellular miRNAs, either free or vesicle-bound, are protected from degradation by binding to Ago2 [44], as both types of miRNAs are sensitive to protease treatment of plasma, but not to RNase digestion [38].

Several types of EVs have been described, including: exosomes, 50–100 nm in diameter and released by exocytosis; microparticles (MPs), 100–1000 nm in diameter and generated by cell membrane shedding; and apoptotic bodies, larger than 1000 nm in diameter and formed during end-stage apoptosis [45,46], thus containing several cellular organelles, such as mitochondria, Golgi and nuclei. Most likely, EV sub-types constitute a heterogeneous mixture of different origins, sizes and constituents. Whereas in healthy individuals, miRNAs appear to circulate mainly associated with Ago2 [38], the scenario is more complex in pathophysiological states, although not yet completely understood. miR-122, for instance, is mostly found in Ago2-free forms in the serum of NAFLD patients [47], namely in exosomes and microparticles [48]. The type of liver injury also appears to dictate the compartmentalization of miRNAs; miR-122 has been found to be predominantly incorporated in exosomes in models of alcoholic liver disease, but is mostly found in exosome-free fractions in acetaminophen (APAP)-induced liver injury [49]. Interestingly, hepatocytes can be both a source and a target for EV-mediated intercellular communication [50]. Indeed, miRNA-carrying EVs are thought to contain specific messages for target recipient cells. Exosomes, for instance, are transferred from body fluids into cells, in a cell-specific manner, upon which the carrying mRNA is able to be translated [51]. HDL-associated miRNAs can also be transferred into cells and be translated. This has been demonstrated in liver cells by Vickers *et al.*, who incorporated native HDL with exogenous miR-375 and miR-223 before incubation with HuH7 cells, leading to a >10 and 250-fold increase of each miRNA intracellular levels, respectively [39]. Further, overexpressed miRNAs were functional, indicating that HDL mediates intercellular communication through the transport and delivery of extracellular miRNAs. Of note, in addition to the classic mRNA-targeting function of miRNAs, a recent study demonstrated that exosomal miRNAs, in this case miR-21 and miR-29a, may also act as ligands that bind to toll-like receptors (TLRs) and activate immune cells [52]. Overall, unique exosomal miRNA expression patterns have been observed in different liver diseases and/or changing with liver disease severity or progression [53], thus reinforcing their potential as diagnostics biomarkers. In particular, EVs have been identified as potential noninvasive markers of disease severity and activity in chronic hepatitis C, NAFLD and cirrhosis [54]. Importantly, the pool of RNA being transported appears to be specific, rather than a random sample of cellular RNA content from the originating cell [55]. For instance, specific sequences present in select miRNAs appear to guide their incorporation into exosomes. Alternatively, sorting of exosomal miRNAs may also be controlled by different enzymes or proteins [56]. Of note, multiple selective and active mechanisms are involved in the release of microRNAs from cells, in comparison to a smaller number of passive release mechanisms [38]. This specific, controlled discharge strengthens the notion that released miRNAs are involved in regulatory and pathophysiologic mechanisms. Although the exact mechanisms controlling this release, as well as the signaling properties elicited by these miRNAs, are still in need of elucidation, their application as putative disease biomarkers is growing.

Profiling of miRNAs in the blood is a multi-step process ranging from sample collection, RNA extraction and quantification, to RNA detection. Although miRNAs are less sensitive to differences in sample handling and processing, as compared to mRNAs or proteins, several reports have demonstrated that differences in the quantity and quality of the isolated miRNAs are to be expected based on which extraction method is used [57,58,59]. As such, and at the moment, there is a lack of standardized and homogenized procedures for miRNA isolation from plasma, with consistency being a key issue. The same is true for detection and profiling methods, the most common being microarrays, quantitative real-time PCR (qPCR) and next-generation sequencing (reviewed in [60,61,62,63,64]). Microarray and next-generation sequencing analyses are useful for high-throughput analysis or discovery of unknown targets, while qPCR is easily and quickly performed, constituting the most commonly-used method for miRNA profiling in the plasma [65,66]. Indeed, it appears that qPCR platforms may be superior to array technologies regarding miRNA profiling from body fluids [67]. Still, and despite its specificity, the sensitivity of qPCR for miRNAs remains a challenge, greatly due to low amounts of RNA recovered from plasma with currently available methods and the lack of proper endogenous controls for normalization. While the use of spiked-in control miRNAs added to serum samples prior to RNA extraction may be helpful in normalizing for isolation differences, absolute quantitative PCR may constitute a more accurate approach [68]. Of note, the fact that circulating miRNAs are found associated with EVs and several different proteins may further contribute to bias when analyzing and interpreting profiling results. As such and to really push the development of miRNAs as biomarkers for liver disease a step forward, future studies should focus on absolute, well-controlled quantification of both Ago/HDL-bound and EVs-associated miRNAs in the plasma of a large cohort of liver disease patients.

## 4. Putative Circulating miRNAs in NAFLD to HCC Progression

Patients with NAFLD are at an increased risk of HCC, both in the presence or absence of cirrhosis [69]. Still, mechanisms connecting both pathologies are not well understood. miRNAs are critical regulators of liver homeostasis, targeting pathways intrinsically associated with metabolism, inflammation, epithelial-to-mesenchymal transition (EMT) and fibrosis, as well as cell death and proliferation. As such, their functional role has been extensively explored in both NAFLD and HCC. Of note, several miRNAs implicated in NAFLD are also altered in hepatic carcinogenesis, although the extent to which these miRNAs promote progression of NASH towards HCC is still under study. Nevertheless, several reports have also been profiling and exploring the roles of these same miRNAs in circulation, and it is now possible to start identifying putative miRNAs that may serve as diagnostic or prognostic markers for disease progression. An overview of the most prominent circulating miRNAs found to be aberrantly expressed in both NAFLD and HCC (Table 1) is given below.

### 4.1. miR-122

miR-122 is the most abundant miRNA in the adult human liver, being primarily expressed in hepatocytes. Because miR-122 directly and indirectly regulates the expression of multiple targets involved in liver homeostasis, its expression has been found abnormal in most, if not all, liver diseases. In particular, miR-122 regulates hepatic lipid metabolism in the liver, including cholesterol biosynthesis [70]. Liver miR-122 expression is strongly decreased in NASH patients compared to steatosis patients and healthy individuals [47,71]. Reduced expression of miR-122 correlates with altered expression of lipid metabolism gene targets in NASH patients [71]. In contrast, serum levels of miR-122 are increased in NAFLD patients, in parallel with circulating ALT levels, liver fibrosis and inflammation [47,72,73]. Accordingly, circulating and hepatic levels of miR-122 also display an inverse pattern on dietary animal models of NAFLD, correlating with the severity of liver histopathology [74,75,76,77]. Of note, elevated circulating levels of miR-122 have been positively associated with obesity and insulin resistance in young adults [78]. Pirola and colleagues also showed that miR-122 mostly circulates in Ago2-free complexes in NAFLD patients and that its serum levels may constitute a better predictor of NAFLD severity when compared to classical disease biomarkers, including serum liver transaminases and caspase-cleaved cytokeratin-18 fragments [47]. In agreement, miR-122 has also been found increased and mainly encapsulated in EVs in experimental NASH, while it was primarily associated with Ago2 proteins in control mice [48]. Finally, it appears that the use of miR-122 integrated within a panel, in this case miR-122-5p, miR-1290, miR-27b-3p and miR-192-5p, may possess a greater clinical value in diagnosing NAFLD, as it was a more sensitive and specific biomarker for NAFLD than ALT and Fibrosis-4 (FIB-4) score [79].

miR-122 has also been found underexpressed in advanced fibrosis [101], as well as in HCC tissues, including advanced tumors with poor prognosis [102,103]. In fact, miR-122 is considered to be a tumor suppressor miRNA, with recent studies showing that mice lacking miR-122 develop normally, but quickly progress into steatohepatitis, fibrosis and HCC [104,105]. Moreover, in animal models of NASH associated with progressive disease resulting in HCC, a reduced expression of miR-122 has been observed at early stages of hepatocarcinogenesis [76,106]. Similarly to NAFLD patients, serum miR-122 levels are increased in HCC and chronic viral hepatitis patients [80,81,82], where it might help to differentiate early HCC from dysplastic nodules (in chronic hepatitis B patients) [83].

Overall, liver miR-122 deregulation appears to contribute to NAFLD progression towards HCC, with circulating miR-122 levels correlating with disease stages and, as such, constituting a likely biomarker of NASH-HCC progression. Still, more longitudinal studies are needed, including in humans. In addition, miR-122 has also been suggested as a potential biomarker of general liver injury, including viral-, alcohol- and drug-related hepatic disease [49,107], while a recent study reported that elevated serum miR-122 levels are associated with a higher overall survival rate in HCC patients [108]. As such, whether miR-122 may be useful as a sole extrahepatic fingerprint for the diagnosis of both NAFLD and HCC also needs to be more systematically evaluated.

### 4.2. miR-21

We have shown that liver miR-21 expression progressively increases with NAFLD disease progression, playing a pathogenic role through targeting of peroxisome proliferator-activated receptor-α (PPAR-α) [109]. Of note, miR-21 has recently been proposed as a potential link between NAFLD and HCC via modulation of the HBP1-p53-Srebp1c pathway [110]. Interestingly, Yamada *et al.* showed that miR-21 serum levels were significantly higher in men with NAFLD than in healthy controls, while no statistical difference was observed in female participants [73]. Further, Cermelli *et al.* reported that circulating levels of miR-21 were unchanged between NAFLD patients and controls [72], and a more recent report stated that circulating miR-21 levels are lower in NAFLD patients [85]. Contradictory findings have also been reported regarding the circulating levels of miR-21 in HCC patients, probably reflecting different etiologies. For instance, Tomimaru and colleagues showed that serum levels of miR-21 are significantly upregulated in HCC patients, correlating with its liver tumoral tissue expression and decreasing post-HCC resection [111]. In contrast, Qi *et al.* reported that miR-21 is downregulated in the serum of patients with HBV-related HCC [80]. In addition, serum miR-21 levels are also increased in patients with different liver diseases, namely chronic viral hepatitis, as well as in other human cancers, such as gastric and colorectal cancer [112,113]. In fact, although increased expression of miR-21 may not be specifically associated with HCC alone, a recent study showed that circulating miR-21 may serve as a potential co-biomarker for early-stage HCC diagnosis [114]. Tomimaru *et al.* also suggested that the combination of plasma miR-21 and AFP has a better differentiating power than miR-21 or AFP alone in discriminating HCC from healthy volunteers [111]. Finally, recent studies suggest that the sensitivity for detecting HCC could be improved by analyzing exosomal miR-21, as miR-21 is enriched in serum exosomes [84].

### 4.3. miR-34a

We and others have shown that hepatocyte cell death, oxidative stress and sustained inflammation constitute key pathogenic mechanisms driving the progression of benign fatty liver towards NASH and, ultimately, hepatic fibrosis and/or liver cancer [115,116,117]. In turn, miR-34a targets proteins involved in regulating cell death, oxidative stress and metabolism. Indeed, miR-34a is found upregulated in the liver of NAFLD patients and reflects histological steatohepatitis severity [71,118]. Concomitantly, we demonstrated that sirtuin 1 (SIRT1), a metabolic target of miR-34a, decreases with disease severity, while hepatic p53 acetylation increases from steatosis to less and, further, more advanced NASH [118]. miR-34a is also found upregulated in the serum of NAFLD patients [72,73]. However, while some authors reported a correlation between human and mouse NAFLD histologic severity and circulating miR-34a levels [72,75], others showed that circulating miR-34a was not increased in a severity-dependent manner when diagnosis was based on ultrasound determination of liver fat [73]. In parallel, miR-34a is also found upregulated in the serum of chronic hepatitis C patients [72]. By promoting apoptosis and cellular senescence, miR-34a also acts as a key regulator of tumor suppression. In agreement, miR-34a has been found downregulated in human HCC tissues compared to adjacent non-neoplastic tissues and is associated with metastasis and tumor invasiveness, representing an independent and significant predictor of patient prognosis when compared to other available clinical parameters [119,120]. Contradictorily, miR-34a was found consistently upregulated in the liver of a mouse model of NAFLD-related hepatocarcinogenesis [76]. In addition, miR-34a was increased in HCC tissue as compared to adjacent tissue in a chemical-induced HCC F344 rat model, while its circulating levels progressively increased with the advancement of hepatocarcinogenesis [86]. Nevertheless, there is no data about the circulating levels of miR-34a in human hepatocarcinogenesis. Still, because increased levels of circulating miR-34a parallels both experimental NAFLD and HCC progression, its potential as a biomarker of human disease severity deserves further elucidation.

### 4.4. miR-16

miR-16 directly represses the expression of several anti-apoptotic genes. In obese patients with NAFLD, histologic NASH positively correlates with the expression levels of pri-miR-16-2 [121]. Interestingly, a study by Cermelli *et al.* showed increased miR-16 circulating levels in NAFLD patients with simple steatosis, which would suggest serum miR-16 as a likely biomarker of early disease. However, in the same study, miR-16 was also found to increase in chronic hepatitis C patients [72]. Nevertheless, plasma miR-16 levels tended to decrease with NAFLD progression, and in HCC, serum miR-16 is downregulated, particularly in patients with tumors over 5 cm in diameter, and correlates with quantitative clinical features, such as platelets and bilirubin [87]. In fact, an earlier study had already reported that lower serum levels of miR-16 could act as a biomarker for HCC, with higher sensitivity than AFP, lens culinaris agglutinin-reactive AFP (AFP-L3%) and des-γ-carboxy prothrombin (DCP). The authors also reported that a combination of miR-16 with AFP, AFP-L3% and DCP offered better-combined sensitivity and specificity for patients with smaller tumors [88]. Finally, a recent report showed that circulating miR-16 levels are significantly lower in hepatitis C virus (HCV)-induced HCC patients, compared to HCV alone, and that the combination of serum miR-16 with serum AFP could discriminate between the two patient populations [89]. Altogether, while miR-16 alone or in combination appears to have great potential as an HCC biomarker, more studies are needed to elucidate whether it could also serve as a diagnostic marker for NAFLD-HCC progression.

### 4.5. miR-192 and miR-375

A recent study has shown that, in parallel with miR-122, miR-192, a target of TGFβ1, and miR-375, a key regulator of glucose homeostasis, are overexpressed in the serum of NAFLD patients, compared to controls [47]. Importantly, both were associated with histological disease severity, further increasing in the serum of NASH patients, compared to simple steatosis patients. All correlated with the NAS score and hepatocyte ballooning, with miR-192 also positively correlating with serum CK-18 levels. To our knowledge, this is the first report addressing the role of miR-375 as a putative biomarker of NAFLD progression. miR-192 circulating levels had been previously described as increased in human patients [79] and in experimental NASH [75]. Moreover, Pirola *et al.* showed a significant association between serum levels of miR-192 and both NAS score and cellular ballooning [47]. Curiously, miR-375 is significantly underexpressed in the liver of NASH patients [71] and in HCC tissues [122,123]. In that regard, yes-associated protein (YAP) and astrocyte elevated gene-1 (AEG-1), whose expressions are suppressed by miR-375, are potent oncogenes overexpressed in the HCC tissue and act as independent prognostic risk factors [122,123,124]. As such, miR-375 may constitute an important molecular link between insulin resistance and hepatocarcinogenesis. In turn, increased miR-375 serum levels have been suggested to act as an excellent biomarker for not only HBV-positive HCC [125], but also for HCC from different etiologies [92]. miR-192 is also upregulated in HCC; Yang *et al.* showed that miR-192 was overexpressed in tumor tissues obtained from early and late recurrent HCC patients post-resection [126]. Further, it may constitute a potential biomarker to differentiate HCC patients from healthy and cirrhosis patients [92], in both HBV-related HCC [90,127] and HCV-related HCC [91].

### 4.6. miR-10b

miR-10b represents an active player promoting liver steatosis by targeting PPAR-α [128]. As it is found overexpressed in steatotic hepatocytes, it promotes the accumulation of intracellular lipids and triglycerides. In a small cohort of NAFLD patients, miR-10b serum levels were found decreased, compared to controls, and inversely correlated with the degree of liver inflammation [93]. miR-10b is overexpressed in HCC and though to promote HCC cell migration and invasion through the homeobox D10 (HOXD10)/ras homolog family member C (RhoC)/urokinase receptor (uPAR)/matrix metalloproteinases (MMPs) pathway [129]. In the serum, miR-10b levels were shown to progressively increase from healthy controls to chronic liver disease patients and, further, to HCC patients [94], highlighting its potential as a noninvasive biomarker for HCC. If circulating miR-10b is confirmed as decreased in NAFLD and as progressively increasing with HCC development, it may constitute an excellent biomarker to monitor NAFLD patients for HCC.

### 4.7. miR-155

Hepatic miR-155 expression has been found increased in murine NAFLD, although it appears to have a protective rather than a causative effect on disease development. This may be attributed, at least in part, to its targeting of liver X receptor α (LXRα) [130]. In fact, a recent study showed that miR-155 hepatic overexpression alleviates experimental NASH and identified carboxylesterase 3/triacylglycerol hydrolase (Ces3/TGH) as a novel direct target of miR-155 [131]. miR-155 has been found upregulated in the serum of patients with chronic HCV infection [132] and in the serum of mouse models of alcoholic liver disease and TLR 9 + 4 ligand-induced inflammatory cell-mediated liver damage [49]. Whether its increased expression in the NAFLD liver also correlates with increased plasma levels is still not clear. Interestingly, in an animal model of NASH progressing to HCC in the absence of an exogenous carcinogen, and therefore more closely resembling the natural course of human disease, miR-155 was found to be significantly upregulated at early stages of hepatocarcinogenesis, in parallel with reduced expression of its target CCAAT/enhancer binding protein beta (C/EBPβ). In addition, ectopic expression of miR-155 promoted the growth of HCC cells, while its inhibition hampered cell growth [76]. In HCC, elevated miR-155 has been shown to target suppressor of cytokine signaling 1 (SOCS1), thereby activating *s*ignal transducer and activator of transcription 3 *(*STAT3) signaling and enhancing the expression of MMP9, which results in increased tumor invasiveness [133]. Of note, administration of exosomes containing synthetic miR-155 mimics into miR-155 knockout mice resulted in a rapid increase, followed by clearance of miR-155 in plasma, with subsequent maximum accumulation of miR-155 in the liver, within hepatocytes [134]. This preferential distribution of miR-155 towards the liver highlights its value as a putative therapeutic target, perhaps even more than as a biomarker. In both cases, however, because miR-155 overexpression has a protective role in NAFLD and a tumor-promoting function in HCC, more studies are needed to elucidate the mechanisms by which miR-155 modulates NAFLD-HCC transition and their consequences on its circulating levels.

### 4.8. miR-181a/b

miR-181a was recently shown to negatively regulate SIRT1, thereby modulating hepatic insulin sensitivity [135]. As such, it may constitute an important player during NAFLD triggering and progression. In fact, mice fed with choline- and folate-deficient (CFD) diet for 12 weeks, resulting in a NASH-like phenotype, display a progressive increase of both liver and plasma miR-181a, paralleling the severity of NAFLD-specific liver pathomorphological features [75]. In liver cirrhosis patients, serum levels of miR-181b were found significantly elevated [136]. miR-181a expression is also increased in both human and mouse cirrhotic or HCC liver tissue samples [137]. Particularly, in hepatitis B virus-related HCC, upregulation of miR-181a appears to induce carcinogenesis by targeting E2F5 [138]. Importantly, plasma miR-181a levels have already been studied and suggested as an accurate and noninvasive biomarker for HCC [94] and, altogether, might also act as a putative NAFLD-cirrhosis prognostic indicator.

### 4.9. miR-15b

miR-15b is considered to be a key regulator of metabolism. In the NAFLD liver, modulation of miR-15b may be correlated with the pathogenesis of hepatic insulin resistance, as it directly targets the insulin receptor (INSR) [139]. miR-15b was recently described as upregulated in experimental NASH, in the mice liver, as well as in the serum of NAFLD patients, compared to healthy subjects [95]. In contrast, one study showed that plasma miR-15b levels were downregulated in a mouse model of diet-induced obesity (DIO), but increased when DIO mice were shifted to a low-fat diet (LFD), paralleling the reverse in body weight, adiposity and blood glucose increases observed in these mice [97]. These opposite findings may be attributed to methodological differences or other unidentified variables. As such, miR-15b should be further studied in the NAFLD context, particularly in obese patients.

In HCC, miR-15b expression levels may predict cancer recurrence. Further, miR-15b modulation could constitute an apoptosis-sensitizing strategy for HCC treatment [140]. Indeed, a recent report showed that, despite being increased in HCC tissues, miR-15b acts as a tumor suppressor gene by directly targeting Rab1A, thereby inducing endoplasmic reticulum stress and apoptosis, as well as growth inhibition [141]. miR-15b is also detected at high levels in the serum of HCC patients, being markedly reduced after primary curative hepatectomy [96,98], suggesting that it is tumor derived. A recent study further showed that miR-15b serum levels were significantly increased in patients who developed HCC from a dysplastic nodule [83], highlighting its potential for diagnosing early HCC or even NAFLD to HCC progression.

### 4.10. miR-33a/b

miR-33a and miR-33b (miR-33a/b) repress the expression of the cholesterol transporter ABCA1, a key regulator of HDL biogenesis. Inhibition of miR-33a/b in non-human primates was shown to raise plasma HDL and lower low density lipoprotein (VLDL) triglycerides, validating the therapeutic potential of the pharmacological inhibition of these miRNAs for the treatment of dyslipidemias and other metabolic syndrome-associated pathologies, including NAFLD [142]. Indeed, obese rats on an HFD supplemented with grape seed proanthocyanidin extract were shown to normalize liver overexpression of miR-33a, with concomitant reduction of plasma and liver lipids [143]. Serum levels of miR-33a may reflect its expression in the liver; a recent study has shown that miR-33a is detected in both α- and β-lipoprotein fractions and is elevated in sera from hyperlipidemic individuals, compared to normolipidemic subjects, positively correlating with inflammatory parameters [99]. Interestingly, serum miR-33a levels are also positively associated with the progress of HBV-induced fibrosis [100]. In fact, liver miR-33a increases with the progression of liver fibrosis, targeting PPAR-α and activating hepatic stellate cells (HSCs) via the PI3K/Akt pathway [144].

## 5. Conclusions and Future Perspectives

Histopathological examination remains the classical gold standard method for the diagnosis and grading of NAFLD and HCC. However, liver biopsy is an invasive procedure with potential serious complications. For HCC in particular, serum AFP combined with imaging techniques has been widely employed for diagnosis, although its sensitivity and specificity are relatively unsatisfactory [7]. As such, several recent studies have focused on the identification of novel non-invasive biological markers to accurately identify individuals who are at risk of developing HCC in surveillance programs, including monitoring of NAFLD patients. Increasing evidence highlights that circulating miRNAs’ expression profiles are potentially useful as fingerprints for disease diagnostic and prognostic evaluations. Ultimate biomarkers for clinical use should have a good specificity and sensitivity to correctly evaluate the severity or presence of some disease state. Particularly for the liver, they should be able to assess the degree or severity of hepatic injury and, ultimately, predict the restoration of normal liver function.

In addition, and ideally, they should also be expressed at high levels in patients and be present at a low concentration, or event absent, in healthy individuals. Although the detection of miRNAs in serum or plasma is minimally invasive and much more convenient when compared to histopathological examination, it remains to be clearly demonstrated whether circulating miRNAs can fully incorporate these desired features. On the one hand, serum and/or plasma miRNA expression levels are stable and easily quantitated, making them suitable as biomarkers for clinical settings. On the other hand, there is a lack in standardization of sample collection, ranging from issues like the fasting status of the patient or animal to the actual blood collection procedures, as well as in data normalization and analysis, which may explain, at least in part, inter-study variations. These factors also relate to the current lack of endogenous controls for serum or plasma miRNA or even the amount and quality of the RNA obtained from different extraction methods. Establishment of guidelines for isolation, quantitation and normalization of miRNAs, when considering their use as disease biomarkers, constitutes a necessary and important step to follow. In addition, miRNA expression levels in the serum may also change during the course of several disorders that are not related to hepatic disease. As such, although a single miRNA has the capacity to act as a sole biomarker, the focus on one particular miRNA may still entail difficult interpretations. Combining multiple miRNAs is likely to enhance their sensitivity and specificity as biomarker panels for a more accurate prediction of those at high risk of NAFLD disease progression. In that regard, Zhou *et al.* identified a panel of miRNAs (miR-21, -26a, -27a, -122, -192, -223, -801) in the serum of a large cohort of HBV-related HCC patients that provided high diagnostic accuracy, independent of disease stage [145]. Another novel and promising approach to improve test performance and obtain further specificity is the evaluation of exosome *versus* protein association of miRNAs. For instance, in mouse models of alcoholic liver disease and inflammatory liver injury, circulating miR-122 and miR-155 were found predominantly associated with the exosome-rich fraction, whereas mainly present in the protein-rich fraction in an acetaminophen-induced liver injury mouse model [49]. Furthermore, in a chemical-induced HCC rat model, combinations of serum and exosomal miRNAs, as well as of AFP and exosome levels, had stronger power in predicting early-stage HCC than AFP alone [146]. Circulating miRNAs could also predict other aspects of HCC management beyond diagnosis, namely prognosis and/or the likelihood of recurrence. For instance, the reduction of functional miR-718 expression in exosomes was associated with the recurrence of HCC after liver transplantation, compared to those without recurrence, and was associated with higher tumor aggressiveness [147]. Overall, understanding the exact function and distribution of circulating miRNAs in extracellular compartments appears to be crucial in order to improve the diagnostic and prognostic accuracy of miRNAs as future biomarkers. Last, but not least, the diagnostic and prognostic value of miRNA signatures should be prospectively evaluated in independent cohorts of patients, using established standardized protocols.

Although our understanding of circulating miRNA expression profiles associated with human HCC attributable to chronic viral infections, such as hepatitis B or C, is improving, little is yet known about miRNA expression patterns associated with NASH-related HCC. Most miRNAs found increased in the serum of HBV-/HCV-related HCC are also overexpressed in patients with chronic viral hepatitis alone, which may suggest that these altered miRNAs may be associated with this particular etiologic agent. Still, it could be that a particular circulating miRNA expression profile may constitute a signature of hepatocarcinogenesis irrespective of the etiology. In addition, most studies to date have been conducted in Eastern Asia and especially in China, which accounts for approximately 50% of all cases of HCC worldwide, due to chronic HBV infection [148]. Overall, it is critical to evaluate the specific changes in the circulating expression profiles of microRNAs in patients with NASH- and metabolic syndrome-associated HCC through large-scale, multi-center studies.

In conclusion, although extensive investigation is still necessary to rigorously validate the potential of circulating miRNAs as biomarkers, these are likely to become a powerful, minimally-invasive means of the diagnosis and prognosis of NAFLD progression to HCC.

## Figures and Tables

**Table 1:** Summary of circulating miRNAs aberrantly expressed in both NAFLD and HCC.

miRNA	NAFLD				HCC			
	Expression Changes	Body Fluids	Study Sample		Expression Changes	Body Fluids	Study Sample	
			Human	Experimental			Human	Experimental
**miR-122**	Up	Whole serum	NASH *vs.* SS and HC; SS *vs.* HC: 19 HC, 18 SS, 16 NASH [72] NAFLD *vs.* HC: 311 HC, 73 mild NAFLD, 19 severe NAFLD (ultrasound diagnosis) [73] Obese *vs.* control: 107 normal weight controls, 123 obese patients [78] NAFLD *vs.* HC: 20 HC, 20 NAFLD [79]	Rats on control or HFD for 2, 6 or 10 weeks [74]	Up	Whole serum	HCC *vs.* CHB and HC: 24 HC, 48 HBV, 48 HBV-related HCC [80] HCC and CHB *vs.* HC: 89 HC, 48 HBV, 101 HCC [81] HCC and CHC *vs.* HC: 10 HC, 30 HCV, 30 HCV-related cirrhosis, 30 with CHC-related HCC [82] HCC *vs.* DN and HC, DN *vs.* HC: 19 HC, 30 CHB-related DN, 120 HBV-related early HCC [83]	
		Whole serum and Ago2-free complex	NASH *vs.* SS and HC; SS *vs.* HC: 19 HC, 30 SS, 47 NASH [47]					
		Whole serum and EV		Mice on CSAA or CDAA diet for 4, 8 and 20 weeks [48]				
		Whole plasma		Mice on control or CFD for 12 weeks [75]				
**miR-21**	Up	Whole serum	Men NAFLD *vs.* men HC: 90 HC, 48 NAFLD (ultrasound diagnosis) [73]		Up	Whole serum	HCC *vs.* CH and HC: 50 HC, 30 CH, 126 HCC [111]	
						Whole serum and exosomes	HCC *vs.* CHB and HC: 30 HC, 30 CHB, 30 HCC [84]	
	Down	Whole serum	NAFLD *vs*. HC: 25 NAFLD, 12 HC [85]		Down	Whole serum	HCC *vs.* HC: 24 HC, 48 HBV, 48 HBV-related HCC [80]	
**mir-34a**	Up	Whole serum	NASH *vs.* SS and HC; SS *vs.* HC: 19 HC, 18 SS, 16 NASH [72] NAFLD *vs.* HC: 311 HC, 73 mild NAFLD, 19 severe NAFLD [73]	Mice on control or CFD for 12 weeks [75]	Up	Whole serum		Chemical-induced HCC in rats [86]
**miR-16**	Up	Whole serum	NAFLD *vs.* HC; SS *vs.* HC: 19 HC, 18 SS, 16 NASH [72]		Down	Whole serum	HCC *vs.* HC: 90 HCC, 90 HC [87] HCC *vs.* CLD and HC: 71 HC, 107 CLD, 105 HCC [88] HCC *vs.* HC and CHC: 20 HC, 40 CHC, 40 HCV-related HCC [89]	
**miR-192**	Up	Whole serum	NASH *vs.* SS and HC: 19 HC, 30 SS, 47 NASH [47] NAFLD *vs.* HC: 20 HC, 20 NAFLD [79]		Up	Whole serum	HCC *vs.* cirrhosis and HC:173 HC, 233 cirrhosis, 261 HBV-related HCC [90] HCC *vs.* CHC and HC: 42 HC, 125 CHV, 112 HCV-HCC [91]	
		Whole plasma		Mice on control or CFD for 12 weeks [75]				
						Whole plasma	HCC *vs.* HC: 77 HC, 70 HCC [92]	
**miR-375**	Up	Whole serum	NASH *vs.* SS and HC: 19 HL, 30 SS, 47 NASH [47]		Up	Whole serum	HCC and CHB *vs.* HC: 100 HC, 75 CHB, 18 HBC, 55 HBV-related HCC [125]	
						Whole plasma	HCC *vs.* HC: 77 HC, 70 HCC [92]	
**miR-10b**	Down	Whole Serum	NAFLD *vs.* HC: 20 HC, 20 NAFLD [93]		Up	Whole serum	HCC *vs.* CLD and HC: 50 HC, 31 CLD, 27 HCC [94]	
**miR-181a**	Up	Whole plasma		Mice on control or CFD for 12 weeks [75]	Up	Whole serum	HCC *vs.* CLD and HC: 50 HC, 31 CLD, 27 HCC [94]	
**miR-15b**	Up	Whole serum	NAFLD *vs.* HC: 42 HC, 69 NAFLD [95]		Up	Whole serum	HCC *vs.* non-HCC: 29 CHB, 57 HBV-related HCC [96] HCC *vs.* DN and HC: 19 HC, 30 CHB-related DN, 120 HBV-related early HCC [83]	
	Down	Whole serum		C57BL/6N male mice on SD or HFD for 12 weeks [97]		Whole plasma	HCC *vs.* cirrhosis and HC, cirrhosis *vs.* HC: 31 HC, 29 cirrhosis, 37 HCC [98]	
**miR-33a/b**	Up	Whole serum and lipo-protein fractions	HLG *vs.* HL, HG and normal controls; HL *vs.* HG and normal controls: 5 normolipidemic/normoglycemic subjects, 6 HL, 9 HG, 5 HLG [99]		Up	Whole serum	Correlation with progress of hepatic fibrosis: 39 CHB [100]	

Ago2, argonaute 2; CFD, choline- and folate-deficient; CH, chronic hepatitis; CHB, chronic hepatitis B; CHC, chronic hepatitis C; CLD, chronic liver diseases; CDAA, choline-deficient and amino acid–defined; CSAA, choline-sufficient and amino acid–defined; DN, dysplastic nodule; EV, extracellular vesicles; HC, healthy controls without any evidence of liver disease; HBV, hepatitis B virus; HCC, hepatocellular carcinoma; HCV, hepatitis C virus; HFD, high fat diet; HG, hyperglycemic; HL, hyperlipidemic; HLG, hyperlipidemic/hyperglycemic; NAFLD, nonalcoholic fatty liver disease; NASH, nonalcoholic steatohepatitis; SD, standard diet; SS, simple steatosis.
